# The impact of digital literacy on the urban integration of migrant workers

**DOI:** 10.1371/journal.pone.0334214

**Published:** 2025-10-17

**Authors:** Chuangxin Zhao, Manping Tang, Changxiang Wang

**Affiliations:** 1 School of Finance and Economics, Chongqing Three Gorges University, Chongqing, China; 2 College of Management, Sichuan Agricultural University, Chengdu, China; Bar-Ilan University, ISRAEL

## Abstract

Improving the urban integration of migrant workers is a crucial requirement for advancing people-centered new urbanization. Based on the micro-data of two China Family Panel Study (CFPS) in 2016 and 2018, this paper analyzes both theoretically and empirically the impact of digital literacy on the urban integration of migrant workers. The findings indicate that digital literacy plays a significant role in enhancing the urban integration of migrant workers; This effect is the strongest in terms of economic integration and relatively weak in terms of psychological integration. The results of the quantile regression show that the positive impact of digital literacy is more pronounced for groups with lower levels of urban integration compared to those with higher levels. Mechanism analysis reveals that digital literacy effectively improves urban integration by enhancing information access and facilitating social interactions. Heterogeneity analysis suggests that digital literacy has a more significant impact on the urban integration of low education levels, the new generation, and migrant workers from the eastern region. The conclusion enriches the theoretical research on the integration of migrant workers into urban areas in the digital age, and provides policy references for the government to attach importance to optimizing digital literacy education and further promote the comprehensive realization of the integration of migrant workers into urban areas.

## Introduction

Urbanization is an essential pathway to modernization and a vital driver of economic growth [1]. Since the 18th National Congress of the Communist Party of China in 2012, the Chinese government has been advancing a people-centered new urbanization strategy, resulting in rapid urbanization levels. With the continuous promotion of urbanization, migrant workers are migrating to cities and towns on a large scale [2]. According to the data from the National Bureau of Statistics, the total number of migrant workers in China reached 296 million in 2022, an increase of 3.11 million compared to 2021. Meanwhile, by the end of 2022, the urbanization rate of permanent resident population in China was 65.22%, still trailing behind developed countries. Moreover, the urbanization rate of registered population was only 46.7% in 2021, and the urbanization rate of the permanent resident population was significantly higher than that of the registered population. This suggests that migrant workers’ cross-regional movement does not follow the classic “migration-settlement-urban integration” model but instead resembles a “bird migration” model where workers “move but do not settle” and “settle but do not integrate” into cities [3]. This unstable urbanization often leads to negative consequences, such as migrant workers’ inability to fully enjoy equal welfare and public services compared to urban residents and the potential for psychological issues, like inferiority and anxiety [4]. The essence of new urbanization is its people-centric approach, and the key to advancing this kind of urbanization lies in integrating migrant workers into urban life [5]. Only by realizing their urban integration and transformation into urban citizens can we eliminate these adverse effects and lay a solid foundation for high-quality development in China’s new urbanization and socioeconomic progress [3,6].

The rapid development of digital technology and digital economy can promote the rapid flow of various resource factors and accelerate the integration of various market players, promoting the transformation of contemporary society from a “social society” to a “mobile society” [7]. “14th Five-Year Plan (2021-2025)” emphasizes the need to enhance national digital skills education and training and improve citizens’ digital literacy. Given their lower educational levels and cognitive abilities, migrant workers face numerous challenges in using digital tools and accessing digital information [8]. If their digital literacy level can be effectively improved, digital technology can play a handy role in their daily work and life, and psychological and social interactions can be driven through digital interaction, it will definitely play a “catalyst” for the urban integration of migrant workers [9]. As internet penetration increases, China’s digital divide has shifted from an “access divide” to an “operation divide”, particularly for migrant workers engaged in non-agricultural work, who need to improve their digital literacy to adapt to digital life and work [10,11]. Enhancing digital literacy is a key factor in bridging the digital divide, which is one of the factors affecting the urban integration of migrant workers [12]. Therefore, an in-depth analysis of the relationship between digital literacy and rural migrant workers’ urban integration not only helps to enrich the relevant theories of digital literacy, but also provides an important reference for exploring new ways to improve the level of rural migrant workers’ urban integration.

With the popularization of the internet, there has been a growing interest in studying how internet usage and media dissemination affect social integration. Research has shown that various online courses and resources on the internet provide convenience for people to actively learn, which is beneficial for enhancing their human capital and furthering social integration [13–15]. Many scholars have found that the use of new media can bridge the social distance between new urban immigrants and local residents, enhance the closeness between groups, and immigrants’ adaptation to urban life [16,17]. For example, Zhang et al. (2022) [9] pointed out that information and communication technology plays a role in the social integration of relocated poverty alleviation households through two mechanisms: providing employment information and enhancing self-identity. Essentially, digital literacy reflects an individual’s or group’s capacity to use digital tools and resources appropriately, construct new knowledge, and innovate in media expression [18,19]. Compared to the definition of digital literacy, the measurement of digital literacy is more specific. For example, Luo et al. (2022) [20] divided digital literacy into five dimensions, such as media operation, information awareness, e-commerce cognition, e-commerce technology and information access. Ji et al. (2024) [21] constructed an evaluation index system of digital literacy from five dimensions: digital general literacy, digital social literacy, digital search literacy, digital creative literacy, and digital security literacy. However, although existing studies have explored the impact of media communication and Internet use on social integration, there is a lack of specific empirical studies on the impact of digital literacy on migrant workers. Can digital literacy promote the urban integration of migrant workers? And what are the paths to urban integration? Is there any difference in the impact of digital literacy on the urban integration of different types of migrant workers? Existing studies have not provided clear answers.

This study focuses specifically on the internal migration context, examining how digital literacy influences the relative level of integration within the migrant worker population as they navigate urban life. We define “urban integration” here not as a direct comparison with native urban residents, but rather as a higher degree of socioeconomic adaptation, social connection, and psychological well-being among migrant workers themselves. Crucially, for this study, we conceptualize “urban integration” of migrant workers as a multidimensional process encompassing three distinct yet interconnected dimensions: Economic Integration: Reflecting migrant workers’ foundational stability in urban life; Social Integration: Capturing migrant workers’ social standing and connections in their new urban environment; Psychological Integration: Representing migrant workers’ subjective well-being and sense of belonging in the city. This multidimensional framework acknowledges that integration is not monolithic [22–24]; Migrant workers may advance in one area (e.g., economic) while facing challenges in another (e.g., psychological). Understanding the specific impact of digital literacy across these dimensions is a key objective of this research.

Based on this, this study uses the data of two China Family Panel Studies in 2016 and 2018 to investigate the impact of digital literacy on the urban integration of migrant workers, and further analyzes its internal logic, mechanism of action and differences among different groups. Compared with previous studies, the possible marginal contributions of this paper are as follows: Firstly, taking digital literacy as a research perspective, this paper conducts a multi-level and multi-angle test on the influence direction, degree and path of digital literacy on the urban integration of migrant workers, which provides beneficial enlightenment for promoting the urban integration of migrant workers and promoting the new urbanization strategy. Secondly, the digital literacy assessment framework of migrant workers, which includes two dimensions of “access to digital equipment” and “use of digital skills”, is systematically constructed, and seven indicators are selected to comprehensively measure the digital literacy index of migrant workers, trying to make up for the shortcomings of existing literatures which mostly examine individual digital abilities from a single aspect of digital access. Thirdly, investigating the heterogeneity of the impact of digital literacy on urban integration from the perspectives of age and educational background, offering decision-making references for improving digital literacy among different groups of migrant workers.

## The direct and indirect effects of digital inclusion

Digital literacy not only directly affects urban integration, but also indirectly influences the process of social integration through channels such as information acquisition mechanisms and social interaction mechanisms.

In the digital age, higher digital literacy usually means higher efficiency in searching opportunities and resources, which can empower migrant workers to break through information barriers and is an important force to promote the urban integration of migrant workers [25]. If migrant workers possess good digital literacy, they can more effectively access job opportunities, educational resources, and social services, improving their quality of life and work efficiency. In addition, people with high digital literacy can build a new social circle in their current residence and maintain close contact with the social capital of their home place by using social media and other digital media, which can not only promote migrant workers to establish a stable interpersonal network in the new environment, but also continue to obtain emotional support from the original social circle and seek practical help when needed [26]. In addition, many cities’ transportation systems, health services, and community governance are already highly digitized. High digital literacy can help migrant workers quickly adapt to the complexity of urban life and integrate into the urban environment more conveniently. Therefore, this paper proposes the following hypothesis:

H1: Digital literacy can enhance the level of urban integration of migrant workers.

In modern cities, critical information such as social services, job opportunities and policy announcements are increasingly being delivered through digital media. Migrant workers with high digital literacy can use search engines, government service platforms, social media and other tools to break through the limitations of traditional face-to-face communication mode, significantly improve the efficiency and breadth of information acquisition, and quickly obtain and screen job opportunities that match their skills [27]. This is conducive to broadening the employment information and channels of migrant workers, effectively addressing their employment needs, and promoting their economic integration through improving employment stability [28]. Although migrant workers are limited to their original agricultural income, they can realize their career transformation by obtaining richer social and economic resources in cities and towns [29]; In this process, digital literacy becomes an important tool for migrant workers to search for information and integrate resources. Therefore, this paper proposes the following hypothesis:

H2: Digital literacy enhances the integration level of migrant workers into urban areas by improving their ability to access information.

Digital literacy can effectively reconstruct the social capital of migrant workers at their work sites, increasing their interaction and communication with local residents, thereby facilitating their social integration [3,9]. Migrant workers often become estranged from their hometown connections due to prolonged periods working away from home [30]. At the same time, due to the high frequency of migrant workers changing jobs and frequent changes in the cities they work in, the original social capital often loses its effectiveness in new work places. Therefore, for a considerable period of time, migrant workers have been in the stage of social capital reconstruction [31,32]. The improvement of digital literacy can help migrant workers better adapt to these changes and provide an effective way to reconstruct their social capital [33]. Studies have shown that improving digital literacy has a significantly positive impact on reconstructing social capital. For example, the use of chat software like WeChat, QQ, and other information and communication technologies can shorten social distances, foster interpersonal interactions, and facilitate faster social integration for migrant workers in new work locations [9,34]. Therefore, this paper proposes the following hypothesis:

H3: Digital literacy enhances the integration level of migrant workers into urban areas by promoting interpersonal relationships.

## Materials and methods

### Data sources

The data for this study are from the China Family Panel Studies (CFPS). The CFPS, conducted by the China Social Sciences Survey Center at Peking University, is a nationwide, large-scale, multidisciplinary social tracking survey project covering 25 provinces (regions, municipalities) in China, including individual, family, and community levels. The survey adopts a multi-stage probability proportional to size sampling strategy, with face-to-face interviews as the primary data collection method, supplemented by computer-assisted personal interviewing technology. The project is conducted biennially, with five complete datasets currently available. CFPS records detailed information on family income and expenditure, individual education, health, internet usage, etc. This study selects CFPS data from two periods in 2016 and 2018 because these two waves maintain consistent measurement scales for both dependent and independent variables. It is important to note that the 2020 wave underwent questionnaire restructuring that resulted in the removal of several key variables used in this study’s analytical framework, therefore it was not included in the analysis. The research subjects are individuals with rural household registration engaged in non-agricultural work in cities, aged 16 and above. After multiple screenings and excluding invalid data, 6580 observation data were finally obtained.

### Variable selection

#### Explanatory variable.

Urban integration. As defined in the Introduction, urban integration for migrant workers in this study is conceptualized as a multidimensional construct comprising economic, social, and psychological dimensions [35–37]. Economic integration is the foundation for the survival of migrant workers in urban areas, reflecting their economic status and the fundamental basis for their long-term survival. Having stable income is the key to their long-term survival [38]; In addition, social security is also an important factor affecting economic integration, and signing labor contracts can help improve the level of social security for migrant workers [32]; Therefore, this paper adopts wage income and labor contract as two indicators to measure the economic integration of migrant workers. When migrant workers enter cities and come into contact with new groups of people, their social status will change, and their social status determines their degree of social integration [39]; In addition, establishing sustained social connections with urban residents is a necessary condition for migrant workers to achieve stable urban life [40]; Therefore, this article measures social integration from two aspects: social status and social relations. For migrant workers, life satisfaction reflects their overall contentment with their urban circumstances, closely related to their psychological integration [41]. In addition, psychological integration is not only reflected in the individual’s adaptation and identification of current urban life, but also reflects his positive expectation of future development and stable sense of belonging. Therefore, this paper measures psychological integration from two indicators: life satisfaction and future development confidence. The specific index system is shown in Table 1.

**Table 1:** Measurement system of urban integration.

Dimension	Index	Index interpretation	Mean value	Standard deviation	Minimum value	Maximum value
Economic integration	Wage income	Total annual wage income of migrant workers (logarithm)	10.216	0.907	5.303	13.122
	Labor contract	Whether to sign a labor contract	0.455	0.498	0	1
Social integration	Social status	Social status in the place of relocation	2.741	0.978	1	5
	Social relations	How well connected do you think you are	7.037	1.726	0	10
Psychological integration	Life satisfaction	Satisfaction with your life	3.686	1	1	5
	Confidence in future development	How confident you are about your future development	4.153	0.878	1	5

To comprehensively evaluate the level of urban integration of migrant workers, this paper uses the entropy method to quantify urban integration. The main reasons are as follows: Firstly, entropy method is an objective weighting method, which sets weights according to the difference degree of indicators, effectively avoids the influence of subjective factors on weight setting, and the output results are highly reliable, providing an effective basis for multi-indicator comprehensive evaluation. Secondly, in the face of dimensional differences and index redundancy problems that may exist in the multidimensional integration index system, entropy method can accurately capture the differentiated contribution of each index to the order degree of the system through standardization processing and dispersion analysis, and can retain the information integrity of the original data better than dimensionality reduction methods such as factor analysis.

#### Core explanatory variable.

Digital literacy. To more comprehensively measure the digital literacy levels of migrant workers, this paper constructs an indicator system based on two dimensions: “digital device access” and “digital skill usage” [42–44]. “Digital device access” reflects an individual’s ability to access and use basic digital tools and devices. This is measured by two questions: “Do you use a mobile phone?” and “Do you use a computer to access the internet?” A value of 1 is assigned for a “yes” response and 0 for a “no” response. On the other hand, “digital skill usage” measures an individual’s ability to use the internet in different contexts. It includes five indicators: “frequency of using the internet for learning,” “frequency of using the internet for work,” “frequency of using the internet for socializing,” “frequency of using the internet for entertainment,“ and “frequency of using the internet for business activities.“ These indicators are rated on a 7-point frequency scale (from “never” to “almost every day”), with values assigned from 0 to 6. By incorporating these two dimensions, this study not only examines the access to digital devices but also assesses how effectively individuals acquire, understand, integrate, create, and use digital information in real-world applications, thereby enhancing the theoretical depth of digital literacy [45]. The specific index system is shown in Table 2. This paper still uses the entropy method to measure the digital literacy level of migrant workers.

**Table 2:** Digital literacy indicator system.

Dimension	Index	Index interpretation	Mean value	Standard deviation	Minimum value	Maximum value
Digital device access	Use mobile phone	Whether to use mobile phone	0.999	0.037	0	1
	Use a computer	Whether you use your computer to access the Internet	0.468	0.499	0	1
Digital skill use	Digital learning	Frequency of using the internet for learning	2.479	2.401	0	6
	Digital work	Frequency of using the internet for work	2.903	2.779	0	6
	Digital social	Frequency of using the internet for entertainment	5.246	1.559	0	6
	Digital entertainment	Frequency of using the internet for socializing	4.797	1.777	0	6
	Digital business	Frequency of using the internet for business	2.540	2.004	0	6

#### Control variables.

Referring to the existing literature [46–48], this paper selects control variables from two dimensions: individual and household. The individual dimension involves seven control variables: gender, age, party membership, marital status, health status, education level and psychological resilience. The household level includes three control variables: annual household income, annual household expenditure and total household size. In addition, in order to control province fixed effect and time fixed effect, province dummy variable and year dummy variable are added as control variables.

#### Mediating variables.

Information acquisition capability and human relationships. Information acquisition capability is identified through the survey question: “The importance of the internet for obtaining information.” Human relationships are measured by the question: “Total amount spent on gifts and cash in the past year.” The descriptive statistics of the main variables in this paper are shown in Table 3.

**Table 3:** Descriptive statistics results of the main variables.

Variables category	Variable name	Variable definition	Mean value	Standard deviation	Minimum value	Maximum value
Dependent variable	Urban integration	Calculated according to the entropy value method	0.490	0.339	0.006	0.996
Independent variable	Digital literacy	Calculated according to the entropy value method	0.478	0.310	0.001	6
Control variables	Gender	1 = male; 0 = female	0.598	0.490	0	1
	Age	Actual age of migrant workers (years)	32.020	9.615	16	75
	Marital status	1 = married; 0 = unmarried	0.674	0.468	0	1
	Educational level	1 = primary school and below; 2 = junior high school; 3 = high school, junior college; 4 = college; 5 = bachelor’s degree and above	2.687	1.114	1	5
	Party membership	Whether the migrant worker is a member of the Chinese Communist Party: 1 = yes; 0 = no	0.131	0.338	0	1
	Health status	1 = very unhealthy; 2 = unhealthy; 3= in general; 4 = healthy; 5 = very healthy	3.413	1.049	1	5
	Psychological resilience	Frequency of feeling lonely in the past week (1 = very frequent, 4 = never)	3.508	0.705	1	4
	Household income	Annual household income (log)	10.863	0.848	6.217	14.25
	Household expenses	Annual household expenditure (log)	10.344	0.951	5.707	14.221
	Family size	Actual number of family members (persons)	3.931	2.167	1	15
Intermediate variables	Information acquisition capability	The importance of the internet to your access to information: 1 = very unimportant; 2 = less important; 3 = general; 4 = important; 5 = very important	4.030	1.134	1	5
	human relationships	Total household spending on gifts and gratuities in the last year (logarithm)	7.339	2.340	0	11.696

### Model setup

#### Baseline regression model.

In order to analyze the impact of digital literacy on the level of urban integration of migrant workers, this paper constructs a benchmark regression model as follows:


$$Y_{ijt}=\alpha +\alpha_{1}X_{ijt}+\alpha_{2}Control_{ijt}+\delta_{j}+\delta_{t}+\varepsilon_{ijt}$$ (1)

Among them, the explanatory variable $Y_{ijt}$ indicates the level of urban integration of migrant workers. Tihe core explanatory variable $X_{ijt}$ indicates the level of digital literacy of migrant workers. $Control_{i}$ denotes a series of control variables affecting urban integration. $\delta_{j}$ represents the province fixed effect; $\delta_{t}$ represents the time fixed effect and $\varepsilon_{ijt}$ is the random error term.

#### Mechanism test model.

In order to test the impact mechanism of digital literacy on migrant workers’ urban integration, this paper adopts the stepwise regression method and sets the following intermediary effect model on the basis of formula (1) [49]:


$$M_{ijt}=\delta +\delta_{0}X_{ijt}+\delta_{1}Control_{ijt}+\delta_{j}+\delta_{t}+\sigma_{ijt}$$ (2)

$$Y_{ijt}=\lambda +\lambda_{1}X_{ijt}+\lambda_{2}M_{ijt}+\lambda_{3}Control_{ijt}+\delta_{j}+\delta_{t}+\sigma_{ijt}$$ (3)

In equations (2) and (3), $M_{ijt}$ is a mechanism variable that includes information acquisition ability and interpersonal relationships, δ and λ are constant terms, $\delta_{0}$ is the impact of digital literacy on the mediator variable, $\lambda_{2}$ is the impact of the mediator variable on the integration of migrant workers into urban areas after controlling for the impact of digital literacy, and the coefficient $\lambda_{1}$ is the direct effect of digital literacy on the urban integration of migrant workers, after controlling for the influence of mediator variables; The other variables are consistent with equation (1).

## Empirical analysis

### Baseline regression results

Table 4 reports estimates of the impact of digital literacy on the urban integration of migrant workers. Among them, model (1) only considers the univariate relationship between digital literacy and rural migrant workers’ urban integration, and models (2) and (3) gradually add variables of individual characteristics and family characteristics respectively. The results show that digital literacy has a significant positive effect on the urban integration of migrant workers. As individual and family characteristic variables are introduced step by step, digital literacy still significantly and positively affects the urban integration of migrant workers, which also indicates the robustness of the regression results to some extent. Consequently, theoretical hypothesis H1 is validated by these findings.

**Table 4:** Baseline regression results.

Variables	Model (1)	Models (2)	Models (3)
Digital literacy	0.338***(0.016)	0.297***(0.020)	0.291***(0.019)
Gender		0.007(0.011)	0.006(0.011)
Age		0.001(0.001)	0.001(0.001)
Marital status		−0.001(0.008)	0.001(0.008)
Educational level		0.034***(0.005)	0.032***(0.005)
Party membership		−0.013(0.020)	−0.011(0.018)
Health status		0.012***(0.004)	0.012***(0.004)
Psychological resilience		0.020***(0.006)	0.021***(0.006)
Household income			0.024***(0.006)
Household expenses			0.007(0.006)
Family size			−0.009***(0.002)
Provincial fixed effect	Yes	Yes	Yes
Time fixed effect	Yes	Yes	Yes
Constant term	0.273***(0.010)	0.093***(0.025)	−0.189***(0.041)
R^2^	0.134	0.147	0.153
Observations	6580	6580	6580

*** denote 1% significance levels, and robust standard errors are in parentheses.

Among the control variables, education level is positively correlated with urban integration level, indicating that migrant workers with higher education level have higher urban integration level. The possible reason is that migrant workers with higher education are more likely to master the skills needed in the urban job market, improve their professional competitiveness, and obtain jobs with high stability. There is also a positive correlation between health status and urban integration, indicating that the healthier migrant workers are, the more conducive to urban integration. The possible reason is that migrant workers are mostly engaged in manual labor, and good physical conditions are the basis for their career stability, which directly affects the continuity of income. The influence of mental toughness on urban integration is significantly positive, indicating that the higher the level of urban integration of migrant workers with strong mental toughness. The possible reason is that individuals with strong psychological resilience can effectively alleviate negative experiences such as discrimination and loneliness in urban life, and improve the level of urban integration. The higher the level of household income, the more beneficial it is to improve the level of migrant workers’ urban integration. The possible reason is that economic power enhances migrant workers’ sense of dignity in the city, and thus their social status. In addition, there is a negative correlation between family size and urban integration, indicating that the increase of total family population is not conducive to rural migrant workers’ urban integration. The likely reason is that the increase in family size places greater responsibilities on migrant workers, who need to spend their income on rural household expenses, reducing their ability to accumulate capital in cities.

### The multi-dimensional impact of digital literacy on urban integration

Although the impact of digital literacy on the overall urban integration of migrant workers has been explored in the previous text, the urban integration of migrant workers is a multi-dimensional process, and digital literacy may have different effects at the economic, social and psychological levels. Therefore, understanding how digital literacy affects different aspects of urban integration can provide us with a more systematic perspective and reveal the multi-level role that digital literacy plays in the process of migrant workers’ urban integration. This section will further discuss the differentiated impact of digital literacy on different integration dimensions of migrant workers. These dimension scores are also quantified using the entropy method. The regression results are shown in Table 5.

**Table 5:** The multi-dimensional impact of digital literacy on urban integration.

Variables	Economic integration	Social integration	Psychological integration
Digital literacy	0.388***(0.029)	0.068***(0.012)	0.015***(0.008)
Control variables	Yes	Yes	Yes
Provincial fixed effect	Yes	Yes	Yes
Time fixed effect	Yes	Yes	Yes
Constant term	−0.362***(0.064)	0.137***(0.038)	0.271***(0.049)
R^2^	0.149	0.081	0.158
Observations	6580	6580	6580

*** denote 1% significance levels, and robust standard errors are in parentheses.

Research has found that digital literacy has a significant positive impact on the economic integration, social integration and psychological integration of migrant workers at the 1% level. That is to say, the higher the digital literacy level of migrant workers, the more conducive it is to promoting their economic integration, social integration and psychological integration. In addition, digital literacy has the greatest impact on economic integration and the least on psychological integration. One possible reason is that migrant workers who master digital technology are more likely to find jobs, start online businesses or utilize digital financial tools, all of which directly enhance their degree of economic integration. In comparison, psychological integration involves deeper identity recognition and subjective sense of belonging, and its formation is restricted by more complex structural factors and individual subjective experiences. Therefore, the promoting effect of digital literacy on psychological integration is relatively limited.

### Quantile regression

Although it has been demonstrated in the previous text that digital literacy significantly affects the urban integration of migrant workers, the impact of digital literacy on the urban integration of migrant workers also has marginal increasing or decreasing effects depending on the individual level of urban integration, resulting in differences in the impact of digital literacy on the urban integration of migrant workers at different levels of integration [45]. The OLS estimation model is a mean regression method that cannot effectively observe the differences in the impact of digital literacy on different levels of urban integration groups. Therefore, this paper uses the quantile regression models for further analysis. Quantile regression results are reported in Table 6. The results show that, basically consistent with the results of OLS regression, digital literacy does have a significant positive impact on the urban integration of migrant workers. From 10% to 90% sub-points, the impact of digital literacy on the urban integration of migrant workers is 0.531, 0.304, 0.204, 0.166, and 0.047, respectively, indicating that with the improvement of migrant workers’ urban integration level, the impact of digital literacy on urban integration is decreasing. Specifically, at the lower integration level percentile (such as 10%), the coefficient of digital literacy is as high as 0.531, indicating that for migrant workers in the early stages of urban adaptation, digital skills, as a key tool resource for breaking through information barriers and obtaining basic public services [50], have the most significant marginal utility. As the degree of integration increases (the coefficient of the 90th percentile drops to 0.047), the promoting effect of digital literacy gradually gives way to structural factors such as social capital accumulation and institutional barriers breakthrough. This is consistent with Berry’s theory of social integration stages – when individuals cross the primary adaptation stage, cultural identity and institutional acceptance become deeper barriers to integration [51]. At the same time, this decreasing trend also echoes the phenomenon of “digital dividend convergence” in technology adoption research [52], that is, the empowering effect of digital technology on vulnerable groups is often most prominent in the basic application stage, while higher-order integration requires organizational support and social network collaboration.

**Table 6:** Quantile regression results of digital literacy on urban integration level.

Variables	Q10	Q30	Q50	Q70	Q90
Digital literacy	0.531***(0.006)	0.304***(0.010)	0.204***(0.029)	0.166***(0.029)	0.047***(0.005)
Control variables	Yes	Yes	Yes	Yes	Yes
Provincial fixed effect	Yes	Yes	Yes	Yes	Yes
Time fixed effect	Yes	Yes	Yes	Yes	Yes
Constant term	−0.110***(0.026)	−0.089***(0.032)	−0.362***(0.080)	0.422***(0.072)	0.695***(0.024)
R^2^	0.037	0.034	0.168	0.040	0.021
Observations	6580	6580	6580	6580	6580

*** denote 1% significance levels, and robust standard errors are in parentheses.

### Robustness tests

#### Robustness test 1.

Considering the endogeneity problem. To address the possible endogeneity problem between digital literacy and urban integration of migrant workers, this paper, following the approaches of previous literature [45,53], and use the indicator “Internet use frequency of migrant workers’ families” as a tool variable to test the relationship between the two. The Internet use of family members may directly affect migrant workers’ digital skills acquisition through knowledge transfer, equipment sharing and other channels, and the technology diffusion within the family has strong relevance, which meets the relevance requirements of instrumental variables; At the same time, the Internet use of family members is mainly driven by their internal needs, and has no direct causal relationship with the urban integration behavior of migrant workers, which meets the exogenous requirements of instrumental variables. Therefore, the instrumental variables in this article may be effective and reasonable. Models (1) and (2) in Table 7 report the regression results of the 2SLS model. The endogeneity test shows that there are endogeneity issues in the impact of digital literacy of migrant workers on urban integration. The results of the first-stage regression show that the instrumental variable is significantly and highly correlated with the potential endogenous variable of digital literacy of migrant workers; Furthermore, the first-stage F-statistic is well above 10, indicating that the selected instrumental variable is not a weak instrumental variable and satisfies the validity test. The results of the second-stage regression show that the coefficient estimates of the independent variables are improved compared to the baseline regression, which implies that the positive effect of digital literacy on the urban integration of migrant workers is likely to be reduced if the endogeneity issue is not considered.

**Table 7:** Robustness tests.

Variables	Model (1)	Models (2)	Models (3)	Models (4)	Models (5)
	The first stage	The second stage	Change the measurement method	Exclude some samples	Shrinking tail method
Digital literacy		2.296***(0.521)	2.016***(0.015)	0.283***(0.020)	0.281***(0.019)
Instrumental variable	0.332***(0.078)				
F value	267.80				
Control variables	Yes	Yes	Yes	Yes	Yes
Provincial fixed effect	Yes	Yes	Yes	Yes	Yes
Time fixed effect	Yes	Yes	Yes	Yes	Yes
Constant term	−0.184***(0.056)	−0.163(0.120)	−0.938***(0.065)	−0.193***(0.041)	−0.188***(0.042)
Observations	6580	6580	6580	6580	6580

*** denote 1% significance levels, and robust standard errors are in parentheses.

#### Robustness test 2.

Change the measurement method. In this paper, the core explanatory variable is digital literacy, and the digital literacy used in the benchmark regression is calculated according to the entropy method. In this part, factor analysis is used to re-measure the digital literacy mentioned above to verify the robustness of the results of the benchmark regression. Model (3) in Table 7 represents the regression results using digital literacy and urban integration variables calculated with factor analysis. The results show that after changing the measurement method of digital literacy, the regression coefficient and significance level are basically consistent with the benchmark regression, indicating that the results are robust.

#### Robustness test 3.

Removing some samples. Older migrant workers may have different integration experiences and challenges compared to younger cohorts, potentially skewing the results [54]. Their lower digital literacy levels could disproportionately affect the overall findings, as they may not engage with technology as actively. In view of this, this paper attempts to delete the sample of elderly people over 60 years old and perform OLS regression again. The results are shown in Model (4) in Table 7. The results indicate that after removing the elderly sample, the regression results of digital literacy and the integration of migrant workers into urban areas are basically consistent with the benchmark regression results, and the research conclusion of this article is robust.

#### Robustness test 4.

Tail reduction method. Although the distribution of urban integration of migrant workers in the sample is relatively average, there are still some migrant workers whose integration degree may be too high or too low. In order to exclude the influence of extreme values, this part carries out tail shrinkage processing for migrant workers with the highest and lowest 1% integration level in the sample, and then carries out empirical test again. The results are shown in model (5) in Table 7. According to model (5), the effect of digital literacy on the urban integration of migrant workers has not changed, and it is still significant at the statistical level of 1%, indicating that the baseline regression results are relatively robust.

## Mechanism testing and heterogeneity analysis

### Mechanism test

Based on the previous analysis, this part conducts stepwise regression according to formulas (1), (2), and (3) to verify the mechanism of action. The model results are as follows:

#### Information acquisition capability.

Firstly, formula (2) is used to analyze the impact of digital literacy on migrant workers’ information acquisition ability. According to the estimation results of model (1) in Table 8, digital literacy has a significant positive impact on the information acquisition ability of migrant workers. It shows that digital literacy can improve the information acquisition ability of migrant workers. Secondly, according to the results of model (2) in Table 8, digital literacy still has a significant positive impact on the urban integration of migrant workers after the variable of information acquisition ability is introduced through equation (3), and information acquisition ability has a significant positive impact on the urban integration of migrant workers, and passes the 1% significance level test. According to the mediating effect test procedure, information acquisition ability plays a partial mediating role in the relationship between digital literacy and urban integration of migrant workers.

**Table 8:** Influence mechanisms of digital literacy.

Variables	Model (1)	Model (2)	Model (3)	Model (4)
	Information acquisition capability	Urban integration	Human relationships	Urban integration
Digital literacy	1.163***(0.057)	0.264***(0.019)	0.238**(0.115)	0.180***(0.020)
Information acquisition capability		0.015***(0.004)		
Human relationships				0.005**(0.002)
Control variables	Yes	Yes	Yes	Yes
Provincial fixed effect	Yes	Yes	Yes	Yes
Time fixed effect	Yes	Yes	Yes	Yes
Constant term	2.558***(0.274)	−0.227***(0.042)	0.177(0.542)	−0.190***(0.042)
R^2^	0.171	0.155	0.139	0.154
Observations	6580	6580	6580	6580

** and *** are statistically significant at 5% and 1%, respectively, and robust standard errors are in parentheses.

#### Human relationships.

Firstly, use equation (2) to test the impact of digital literacy on the human relationships of migrant workers. According to the estimation results of model (3) in Table 8, it can be seen that digital literacy has a significant positive impact on the human relationships of migrant workers, indicating that digital literacy helps to promote interpersonal relationships among migrant workers. Secondly, according to the results of model (4) in Table 8, it can be seen that after introducing the variable of human relationships through equation (3), digital literacy still has a significant positive impact on the urban integration of migrant workers, and the variable of human relationships has a significant positive impact on the urban integration of migrant workers, which is tested at a significance level of 5%. According to the testing steps of the mediation effect model, human relationships play a partial mediating role in the relationship between digital literacy and the urban integration of migrant workers.

In summary, digital literacy improves migrant workers’ urban integration by enhancing their ability to obtain information and promoting human relations, and hypothesis 2 and hypothesis 3 are verified.

### Heterogeneity analysis

#### Heterogeneity analysis based on the number of years of education of migrant workers.

According to the difference in years of education, migrant workers are classified high education group (high school and above) and low education group (high school and below) [45]. The results of model (1) and model (2) in Table 9 show that the influence coefficients of digital literacy on the urban integration of migrant workers in the group with high education level and the group with low education level are 0.219 and 0.276 respectively, both of which pass the 1% significance level test, and the empirical P-value of the coefficient difference test between groups is 0.041. It shows that the effect of digital literacy on the urban integration of migrant workers with low education experience is more obvious. The main reason is that the low educated group is hindered by traditional integration paths, and the compensatory effect of digital literacy is more significant: digital tools make up for their shortcomings in information acquisition, skill training, etc., and break through geographical limitations through social networks to reconstruct social capital. The highly educated group has strong adaptability to resources, limited marginal benefits of digital technology, and relies more on existing educational advantages for integration. Therefore, digital literacy has a greater promoting effect on the urban integration of low educated migrant workers.

**Table 9:** Heterogeneity analysis.

Variables	Model (1)	Models (2)	Models (3)	Models (4)	Models (5)	Models (6)
	High level of education	Low level of education	New generation	Older generation	Eastern region	Central and Western region
Digital literacy	0.219***(0.033)	0.276***(0.018)	0.298***(0.017)	0.217***(0.031)	0.273**(0.027)	0.216***(0.028)
Control variables	Yes	Yes	Yes	Yes	Yes	Yes
Provincial fixed effect	Yes	Yes	Yes	Yes	Yes	Yes
Time fixed effect	Yes	Yes	Yes	Yes	Yes	Yes
Constant term	0.053(0.131)	−0.174**(0.076)	−0.146**(0.072)	−0.363***(0.130)	−0.010***(0.021)	−0.217***(0.055)
R^2^	0.096	0.105	0.158	0.148	0.170	0.123
Experience P-value	0.041**		0.004***		0.011**	
Observations	1756	4824	4986	1594	2932	3648

*** and ** represents significant at the 5% and 1% statistical levels respectively, with robust standard errors in parentheses. The “empirical P-value” is used to test the significance of coefficient differences between groups, obtained through bootstrap sampling 1000 times.

#### Heterogeneity analysis based on age of migrant workers.

Due to the differences in cognitive ability and reception ability of migrant workers of different ages [55], this paper examines the heterogeneity of the impact of digital literacy of migrant workers of different ages on urban integration. Divide the sample into two parts based on the age of the respondents. Among them, migrant workers born before 1980 are defined as the older generation of migrant workers, and those born after 1980 are defined as the new generation of migrant workers [54]. The results of model (3) and model (4) in Table 9 show that digital literacy has a significant impact on the urban integration of both the new generation and the old generation of migrant workers, but it has a greater impact on the urban integration of the new generation of migrant workers. It may be interpreted as the new generation of migrant workers growing up in a more technologically advanced environment, generally possesses higher digital literacy and is more adaptable to using digital tools for social integration. Their familiarity with technology enables them to navigate online platforms for information, social connections, and services more effectively. Therefore, for the new generation of migrant workers, digital literacy plays a greater role in promoting their urban integration.

#### Heterogeneity analysis based on regions.

To investigate the impact of regional development differences on the distribution of digital dividends, this paper divides the samples into the eastern region and the central and western regions based on the regional division standards of the National Bureau of Statistics. The results of models (5) and (6) in Table 9 show that the promoting effect of digital literacy on the urban integration of migrant workers in the eastern region is significantly higher than that in the central and western regions. Moreover, the empirical P-value of the inter-group coefficient difference test is 0.011, indicating that there are structural heterogeneous characteristics among regions. The main reason is that the well-developed digital infrastructure and rich application scenarios in the eastern region provide migrant workers with a more frequent digital survival interface, enabling digital literacy to be more efficiently transformed into integration capabilities.

## Conclusion and insights

The social integration of migrant workers in urban areas is a critical manifestation of advancing people-centered new urbanization. In the digital age, digital literacy has profoundly affected people’s lives in various aspects of clothing, food, housing and transportation. For migrant workers, digital literacy can help them integrate into towns and adapt to urban life. However, there is a lack of research on the relationship between digital literacy and urban integration in academia, and insufficient attention has been paid to the accumulation of digital literacy among migrant workers. In view of this, this paper empirically analyzes the impact and mechanism of digital literacy on the urban integration of Chinese internal migrant workers based on CFPS data from 2016 and 2018, focusing on relative improvements within this specific group. The results show that digital literacy has a significant promoting effect on the urban integration of migrant workers. This effect is strongest in terms of economic integration and relatively weak in terms of psychological integration. Moreover, the impact of digital literacy on the integration of migrant workers into urban areas has a marginal diminishing effect. Digital literacy has accelerated the urban integration of migrant workers by enhancing their ability to access information and promoting interpersonal relationships. In addition, the effect of digital literacy on promoting the urban integration of migrant workers is heterogeneous, and the effect of digital literacy on the urban integration of migrant workers with low education level, the new generation of migrant workers, and migrant workers in the eastern region is more obvious. In order to better play the role of digital literacy in promoting urban integration of migrant workers and accelerate the construction of new urbanization, this paper proposes the following countermeasures and suggestions:

Firstly, the government should pay attention to the impact of emerging new resident qualities such as digital literacy on urban integration in the digital age. This study finds that digital literacy has a significant promoting effect on the urban integration of migrant workers, and the marginal effect is even higher for groups with low integration levels. This indicates that policies can prioritize exploring basic digital skills popularization plans covering all migrant workers, especially designing “zero-threshold” training modules for disadvantaged groups in urban integration. Meanwhile, it is suggested that the pilot program incorporate digital literacy into the community public service system. Free equipment borrowing and operation guidance should be provided through grassroots service stations to unleash the maximum empowerment potential of digital technology for vulnerable groups.

Secondly, mechanism tests have confirmed that digital literacy promotes urban integration through both enhancing information acquisition capabilities and expanding social relationships. Therefore, it is suggested to establish the “information-social” dual-wheel driven support system. On the one hand, integrate the entry points of public services such as employment, social security and medical care, and build a digital information platform exclusively for migrant workers to reduce the cost of information search. In addition, encourage communities to promote interaction between migrant workers and citizens through the “online community + offline activities” linkage model.

Thirdly, heterogeneity analysis reveals that digital literacy has a more significant promoting effect on low-education, the new generation, and migrant workers from the eastern region. This suggests that when cultivating the digital literacy of migrant workers, differentiated intervention strategies can be formulated. For groups with low educational attainment and the older generation, basic skills literacy can be piloted. For the new generation, advanced application training can be explored. The eastern region needs to further verify the feasibility of transferring digital infrastructure experience to the central and western regions, and at the same time assess the effect of special transfer payments on enhancing digital inclusiveness in less developed regions.

It is worth noting that this paper still has the following shortcomings: Firstly, in terms of indicator design, the seven indicators of digital literacy in two dimensions and the six indicators of urban integration used in this paper are not sufficient to fully measure migrant workers’ digital literacy and urban integration, and the existing literature cannot fully compensate for this defect. How to establish a comprehensive and reasonable comprehensive index to measure migrant workers’ digital literacy and urban integration is worth further research. Secondly, in terms of research methods, this paper uses empirical methods for research, but considering that urban integration is a multidimensional and dynamic complex process, regression analysis relying on sampling monitoring survey data is difficult to fully capture the underlying mechanisms. Therefore, in the future, representative regions can be selected for longitudinal case studies, and more information that is difficult to discover through questionnaire surveys can be obtained through in-depth interviews, thereby providing policy makers with richer practical basis. Thirdly, our measure of “urban integration” is defined and assessed relative to the experiences and outcomes within the migrant worker population itself. While this provides valuable insights into the factors driving differential integration success among migrant workers, it does not directly compare their levels of economic attainment, social status, or psychological well-being to those of native urban residents. Future research incorporating a native comparison group could offer an even more comprehensive understanding of the absolute integration gap and the specific role digital literacy might play in bridging it. However, the focus on internal migration within China presents a significant strength. By examining a population largely sharing a common national language and cultural background, this study minimizes confounding factors like language barriers and major cultural differences that often complicate studies of international migration. This allows for a “cleaner” analysis of how digital literacy, as a specific set of skills and resources, influences the adaptation process within a relatively homogeneous linguistic and cultural context.

## Supporting information

S1 File Empirical dataset for “The impact of digital literacy on the urban integration of migrant workers”. This file contains the anonymized micro-level dataset and the corresponding codebook used for the empirical analysis in this study. The data includes all variables employed in the regression models, such as the comprehensive urban integration index, digital literacy index, control variables (e.g., gender, age, education, household income), and mediating variables.
(XLSX)
